# Recurrence Yield of Stereotactic Biopsy of Suspicious Calcifications After Breast Conservation Therapy

**DOI:** 10.7759/cureus.24318

**Published:** 2022-04-20

**Authors:** Javaria Aleem, Sara Rehman, Mehreen Shafqat, Hamd Zahra, Javeria Ashraf, Imran Khalid Niazi

**Affiliations:** 1 Department of Radiology, Shaukat Khanum Memorial Cancer Hospital and Research Centre, Lahore, PAK; 2 Department of Radiology, University Hospitals of North Midlands NHS Trust, North Midlands, GBR

**Keywords:** tumor resection margins, suspicious calcifications, recurrence, breast cancer, microcalcifications, mammogram, stereotactic core biopsy, breast conservation surgery

## Abstract

Aim

To analyze the histopathological outcome of stereotactic biopsies of newly developed suspicious calcifications at lumpectomy scar site in patients with breast conservation surgery (BCS) to determine the incidence of malignancy and the association of mammographic appearance of recurrent microcalcification and their distribution. We also determined the association of disease recurrence with the presence of calcifications in original tumor and lumpectomy resection margins with the risk of recurrence.

Materials and methods

This study is a retrospective review of mammograms of patients with breast cancer from 2010 to 2021 who underwent stereotactic biopsy of newly developed suspicious calcifications at scar site appreciated on annual follow-up mammogram after breast conservation surgery (BCS) with no mass on correlative ultrasound. The radiological and pathological features of the patients' primary tumor and new calcifications were obtained from the hospital's electronic patient record system.

Results

A total of 84 patients with breast cancer developed suspicious microcalcifications at the lumpectomy scar site detected on follow-up mammograms after BCS, and 28.6% showed malignant histopathological outcomes. All malignant cases demonstrated pleomorphic morphology. All amorphous (9.5%) and coarse heterogeneous (54.8%) calcifications were benign. The distribution pattern of recurrent malignant calcifications was grouped in 9.5%, regional in 2.4%, linear in 9.5%, and segmental in 7.1%. Calcifications in primary tumors were found in 20.2% of cases. Positive margins were found in 7.1% of these malignant cases. Statistically, there was a strong association between calcification morphology, calcification distribution, presence of calcifications on baseline mammogram, and tumor resection margins. The presence of calcifications in primary tumors and positive resection margins were identified as significant independent risk factors of malignant recurrent calcifications in the logistic regression model and marginal statistical significance in the multivariable logistic regression (MLR) model.

Conclusion

The interval development of pleomorphic calcifications after BCS with either linear or segmental pattern, positive resection margins, and associated calcifications in primary tumors was related to the increase in the risk of recurrence. Although amorphous and coarse heterogeneous morphology with grouped distribution showed benign outcomes, stereotactic biopsy is recommended to exclude disease recurrence in this high-risk patient population.

## Introduction

Breast conservation surgery (BCS) in the form of lumpectomy and adjuvant radiotherapy is commonly used for early-stage breast carcinoma and has gained increased acceptance all over the world [1]. Physical and mammographic examination is routine for the follow-up of treated patients. The locoregional recurrence rate at the site of previous surgery is 1%-2% per year for 10 years [2,3]. On follow-up after radiotherapy, it is difficult to differentiate between scar tissue and recurrence. Mammography is used to detect recurrence in the form of abnormal mass density, scar thickening, or the development of suspicious calcifications [4]. New developing calcifications at the scar site could be benign dystrophic related to postradiation healing, fat necrosis, or the development of recurrent carcinoma. Occasionally, it is difficult to differentiate between calcifications due to recurrence and those developed due to other benign causes. These patients are candidates for stereotactic core biopsy. The early evaluation of these suspicious calcifications helps in the timely detection and management of recurrent breast carcinoma [5].

The primary aim of our study is to determine the incidence of malignancy in suspicious calcifications identified on mammograms after breast conservation therapy and evaluate the morphology and distribution characteristics. We will discuss the effect of other factors possibly associated with recurrence, such as time from completion of therapy and tumor resection margins after the surgical resection of the primary tumor.

## Materials and methods

Study design and participants

A retrospective data collection and analysis were approved by the institutional review board of our hospital and waived the need for informed consent from all patients (EX-15-04-20-02).

We included all patients with breast cancer undergoing stereotactic biopsy who developed suspicious calcification at lumpectomy scar site on annual follow-up mammogram after BCS with no mass on correlative ultrasound from January 2010 to December 2021. We excluded patients undergoing stereotactic biopsy for suspicious calcifications before surgery, calcifications in contralateral breast on postsurgical mammogram, calcifications at a site other than lumpectomy scar, and distant metastasis at the time of diagnosis.

Image data analysis and reference standards

The hospital information system (HIS) was searched for patients with breast cancer undergoing stereotactic biopsy. In total, 84 patients developed new suspicious calcifications at the lumpectomy scar site after breast conservation surgery and radiotherapy of primary breast cancer. Mammograms were reviewed independently by radiology residents in the fourth year of training (R4) for morphology and distribution of calcifications. Calcification morphology was categorized as amorphous, coarse heterogeneous, and pleomorphic, while the parameters for distribution pattern included grouped, regional, linear, and segmental. These criteria were based on the BI-RADS lexicon fifth edition by the American College of Radiology (ACR) [6,7].

The findings were further independently reviewed by two radiologists who had three years of experience in breast radiology. Any discrepancy was further dismissed by consensus. Demographic, histopathological, and treatment data were also documented. Stereo-guided core biopsy was performed using the GE Medical sonography essential system (GE Medical System SCS, Buc, France). On average, five cores were taken from the site of calcifications.

Statistical analysis

The SPSS version 20 (IBM Corporation, Armonk, NY, USA) was utilized for statistical analyses. Mean and standard deviation/median and range were employed to summarize quantitative data. Frequencies and percentages were used to organize qualitative data. The association of categorical variables was determined using the chi-square test. Univariable and multivariable logistic regression (MLR) model was used to identify the independent risk factors associated with recurrence using a forward stepwise approach. The statistical significance was taken at p<0.05.

## Results

From 2010 to 2021, 354 stereotactic biopsies were performed, of which 84 were performed for new suspicious microcalcifications at the lumpectomy scar site. The mean age of patients was 52 years. Out of 84 (100%) patients, 24 (28.6%) were malignant on stereotactic specimen histology, and 60 (70%) patients were benign. The average time of the development of calcifications in the treated breasts after therapy (lumpectomy and adjuvant chemoradiotherapy) was 26 months. Twelve (50%) cases developed malignant recurrent calcifications within 12 months of the completion of therapy. No significant correlation was found between the time of the recurrence of malignant calcifications and the completion of therapy (p=0.319). Table 1 shows the histology of stereotactic biopsies of new pleomorphic calcifications at the lumpectomy scar site. Of the 24 malignant cases, six (25%) were found to be invasive ductal carcinoma, 10 (42%) were ductal carcinoma in situ (DCIS), two (8.3%) were invasive ductal carcinomas with DCIS, and six (25%) had lobular carcinoma in situ (LCIS).

**Table 1:** Histopathology of recurrent malignant calcifications after breast conservation surgery.

Histopathology	Malignant calcification (n=24)
Invasive ductal carcinoma	6 (25%)
DCIS	10 (42%)
Invasive ductal carcinoma with DCIS	2 (8.3%)
LCIS	6 (25%)

**Figure 1:**
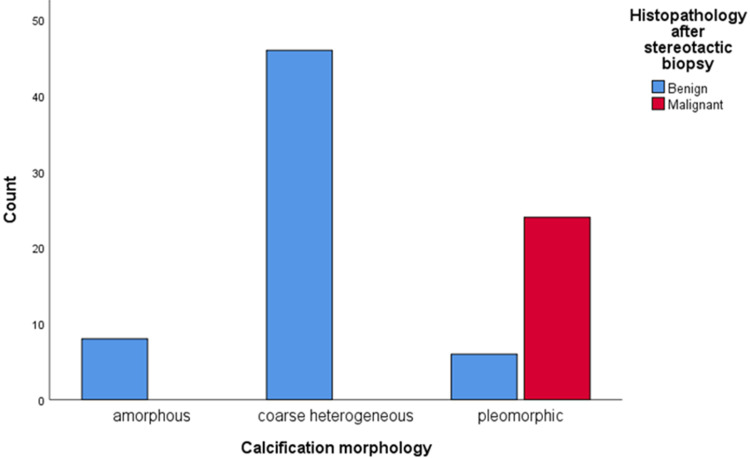
Association of calcification morphology with tumor recurrence.

**Figure 2:**
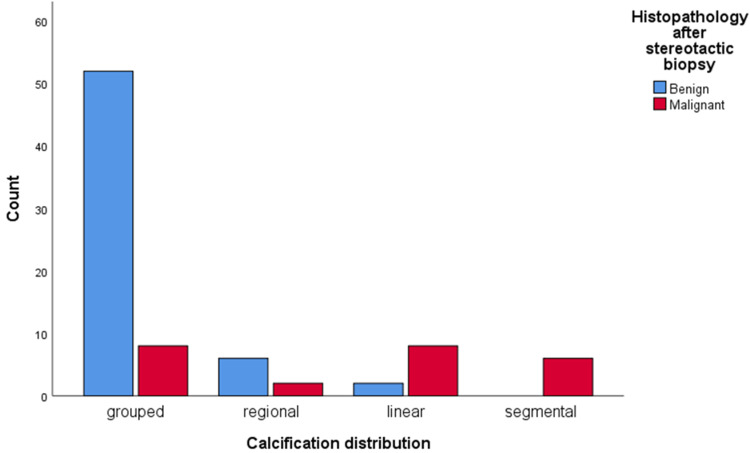
Association of calcification distribution with tumor recurrence.

The relationship between the appearance of microcalcifications and the histology of stereotactic biopsies is shown in Table 2. All 24 (28.6%) malignant cases showed pleomorphic morphology of calcifications, while benign calcifications had amorphous (9.5%) and coarse heterogeneous (54.8%) morphology (Figure 1). The distribution pattern of these 24 malignant cases was grouped in eight (9.5%) patients, regional in two (8.3%), linear in eight (9.5%), and segmental in six (7.1%) patients (Figure 2). There is a statistically significant association between calcification morphology and distribution with the development of malignant scar site calcifications (p=0.001 each).

**Table 2:** Relationship between calcification morphology and distribution with the histopathology of stereotactic biopsies of new microcalcifications at the lumpectomy scar site.

Parameters	Total (n=84) (100%)	Benign (n=60) (100%)	Malignant (n=24) (100%)	p-value
Calcification morphology				0.001
Amorphous	8 (9.5%)	8 (9.5%)	-	
Coarse heterogeneous	46 (54.8%)	46 (54.8%)	-	
Pleomorphic	30 (35.7%)	6 (7.1%)	24 (28.6%)	
Calcification distribution				0.001
Grouped	60 (71.4%)	52 (61.9%)	8 (9.5%)	
Regional	8 (9.5%)	6 (7.1%)	2 (2.4%)	
Linear	10 (11.9%)	2 (2.4%)	8 (9.5%)	
Segmental	6 (7.1%)	-	6 (7.1%)	

Of 24 cases that developed malignant calcifications, 17 also had malignant calcifications in the original tumor on baseline mammogram. There is a statistically strong association between the presence of calcifications in the original tumor and the recurrence of calcification after therapy (p=0.05). In addition, 18 (75%) malignant cases had clear resection margins on post-lumpectomy histology specimens, while resection margins were involved and re-excision was attempted in six (7.1%) cases. Similarly, there is a strong association between involved resection margins and tumor recurrence (p=0.029). These two variables were also identified as significant independent risk factors of malignant recurrent tumors in the logistic regression model. In the multivariable logistic regression model, calcification on baseline mammogram (original tumor) was present (adjusted odds ratio (AOR): 7.50; 95% confidence interval (CI): 1.25-44.78; p=0.03), and resection margins on lumpectomy specimen also show marginal statistically significant relationship toward recurrent malignant diagnosis (AOR: 4.66; 95% CI: 1.18-18.40; p=0.03) (Table 3). This means that the presence of calcifications in the original tumor and involved resection margins increases the risk of recurrence in BCS.

**Table 3:** Risk factors of recurrence using the logistic regression model.

Variable categories	Univariable logistic regression model odds ratio (95% confidence interval), p-value	Multivariable logistic regression model odds ratio (95% confidence interval), p-value
Calcifications on baseline mammogram (original tumor)	-	-
Absent	Reference	Reference
Present	2.80 (1.01-7.76), 0.04	2.56 (0.10-7.30), 0.07
Resection margins	-	-
Not involved	Reference	Reference
Involved	4.66 (1.18-18.40), 0.03	4.20 (1.03-17.11), 0.04

## Discussion

Breast conservation surgery is a reasonable treatment option for selected patients with breast cancer [8,9]. The survival of women treated with breast conservation therapy is similar to mastectomy. The reported five-year survival rate for BCS is 92.9%, while for mastectomy is 89.7% [10]. However, the risk of recurrence is still present [11]. The rate of local recurrence in women treated with breast conservation therapy is reported to be 5%-10% in the first five years, while 10%-15% after 10 years of treatment [12,13]. In our tertiary care cancer hospital, imaging, histopathology, and other risk factors of all new patients with breast cancer are discussed in a multidisciplinary team (MDT) meeting for concordance, and the most appropriate surgical plan, such as BCS versus mastectomy, is then decided.

Survival after local recurrence depends on the stage at which recurrence has occurred. Therefore, the early diagnosis of recurrence is important. Mammography has a valuable role in the early detection of recurrence [14,15]. The mammographic features of recurrent carcinoma include mass density, increased thickness/density of scar, increased skin thickness, and the development of new calcifications. The reported incidence of the development of new calcifications after BCS ranges from 16% to 31% [5,16]. New calcifications at the scar site may represent disease recurrence or posttreatment change. It has been reported that treatment-related calcifications usually develop 2-5 years after treatment [12,17]. The reporting radiologist must be aware of the morphological features of suspicious microcalcifications to differentiate between benign and malignant calcifications. Stereotactic biopsy finds its role in obtaining tissue diagnosis for patients with newly developed calcifications without a corresponding mass on correlative ultrasound.

In the various previous studies, the predictive values for the local tumor recurrence of new suspicious calcifications without an associated mass in the conservatively treated breast range from 33% to 100% [2,5,14,18-21]. Recurrence in form of mammographically detected microcalcifications was found in 43% of cases in a study by Stomper et al. [14]. Malignant calcification developed in 53% of cases in another study [16]. In our study, we evaluated the recurrence of microcalcifications that developed at the lumpectomy scar site only including 28.6% of malignant cases.

In the evaluation of calcifications at the lumpectomy scar site, morphology and distribution patterns are used to determine the probability of malignancy [22]. In previous studies, the incidence of benign outcomes for new calcifications following BCS has been reported to range from 42% to 85% [8,11,12]. In our study, the incidence of benign calcifications was higher than in two previous studies (i.e., 71%) [8,11]. In those studies, stereotactic biopsy was performed irrespective of the morphological features and also for new calcifications developing in contralateral breast or in the same breast away from the scar site, so our results can be different from the earlier studies. In the study by Giess et al., stereotactic biopsy was done for numerous calcifications developing at the scar site with an incidence of benign histopathology accounting for 85% [18].

**Figure 3:**
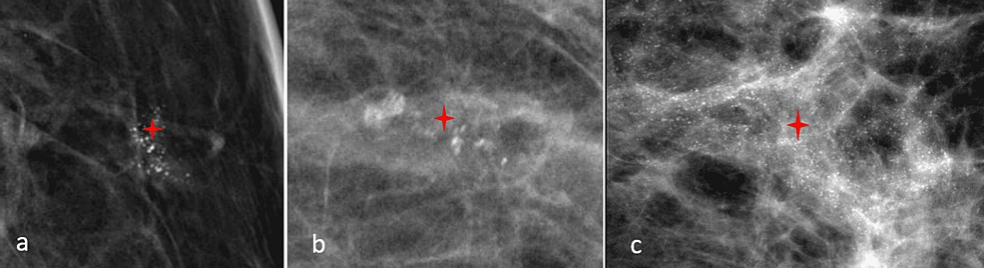
Digital mammographic images showing calcifications with suspicious morphology (red star): (a) amorphous, (b) coarse heterogeneous, and (c) pleomorphic.

In earlier studies, the highest portion of malignant recurrent calcification showed pleomorphic morphology. For reference, in the study of Günhan-Bilgen and Oktay, 46% of malignant calcifications are pleomorphic morphology [5]. In another study conducted by Dershaw et al., recurrence was found in 77%, with pleomorphic morphology [12]. In our study, 30 (35.7%) patients had pleomorphic morphology on mammograms, and of these, 24 (28.6%) patients had malignancy on histopathology. Furthermore, eight (9.5%) patients had amorphous and 46 (54.8%) showed coarse heterogeneous morphology. These calcifications were found to have benign histopathology on the results of stereotactic biopsy. The schematic presentation of calcification morphology is shown in Figure 3.

**Figure 4:**
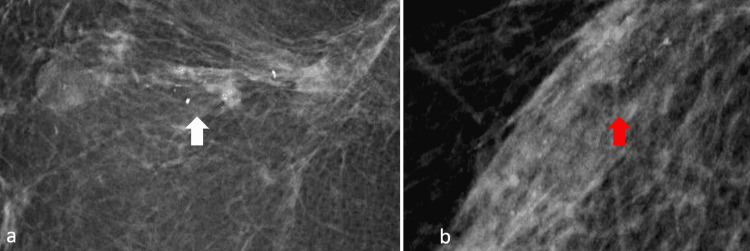
Digital mammographic views showing linear and linear branching patterns of calcifications in two different patients: (a) 44-year-old female four years post-BCS (white arrow) and (b) 46-year-old female two years post-BCS (red arrow). Both were found to have malignant histology following stereotactic biopsy. BCS: breast conservation surgery

**Figure 5:**
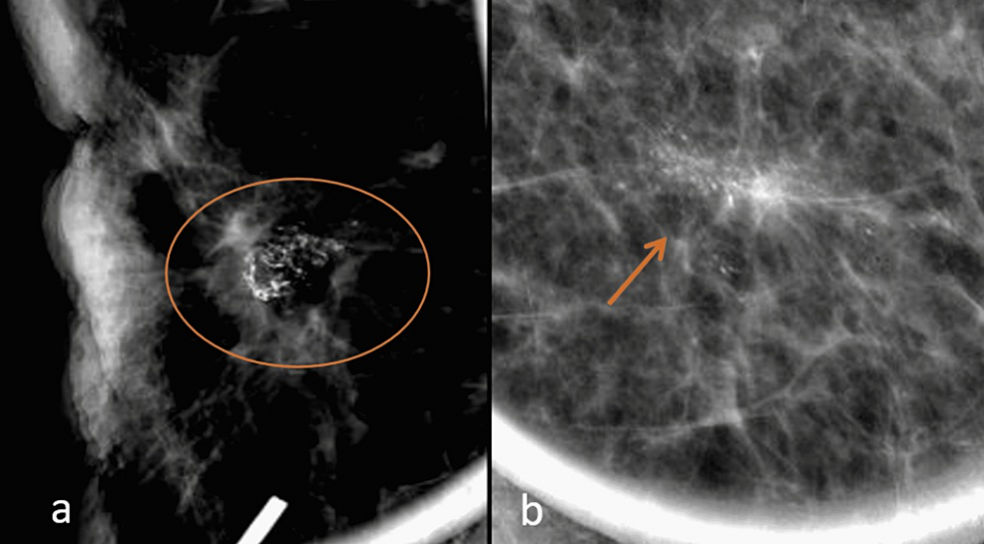
Digital mammographic magnified views of coarse heterogeneous calcifications with grouped distribution pattern in the tumor bed at the scar site in a (a) female five years post-BCS (orange circle) and (b) two years post-BCS (orange arrow). BCS: breast conservation surgery

**Figure 6:**
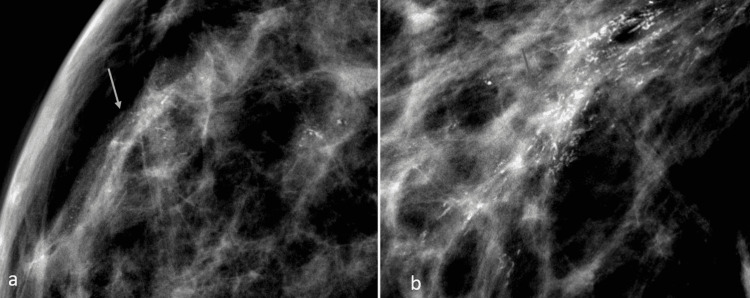
Digital zoom mammographic views of pleomorphic calcifications in a segmental distribution within the right breast tumor bed in a (a) 59-year-old female (yellow arrow) and (b) 60-year-old female (green arrow) post-BCS. BCS: breast conservation surgery

Of the histologically proven malignant calcifications, grouped and linear distribution was observed in 33% (8/24) of cases each, while 25% (6/24) of cases showed segmental distribution. Regional distribution was observed in 8% (2/24) of patients (Figures 4, 5, 6). Most of the histologically proven benign calcifications had grouped distribution patterns (87%). In the study by Günhan-Bilgen and Oktay, most of the patients demonstrated clustered distribution patterns (66%), while linear and regional patterns were observed in 17% of patients [5]. In the study by Dershaw et al., the majority of patients (71%) demonstrated clustered distribution patterns, while linear and segmental patterns were observed in 18% and 12% of patients, respectively [12]. Histopathological correlation with reference to distribution has not been documented in previous studies.

**Figure 7:**
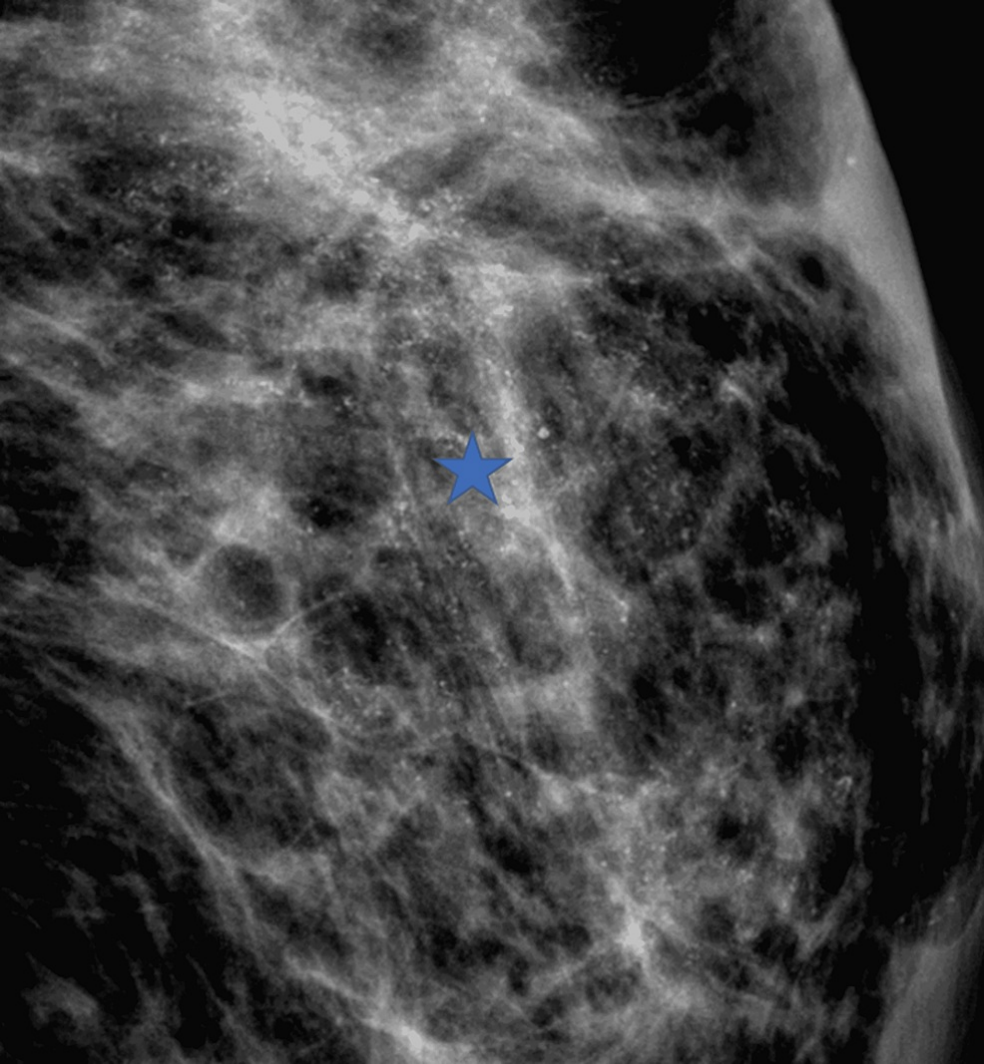
Digital zoom mammographic MLO projection of pleomorphic calcifications in a regional distribution within the tumor bed in a 56-year-old female two years post-BCS (blue star), which were found to be malignant at biopsy. MLO: mediolateral oblique

Previous studies have shown that the risk of malignancy in newly developed calcifications in the treated breast is not influenced by the presence or absence of calcifications in the original tumor [5,18,19,23]. Our study is contrary to this; 71% (17/24) of cases that had developed malignant recurrent calcifications also had calcifications in the primary tumor on baseline mammogram. It has been reported that most patients with DCIS who presented with calcifications in the original mammogram also had recurrences with calcifications [24]. In our study, DCIS was found in 42% of recurrent calcification with pleomorphic morphology and regional distribution pattern (Figure 7).

There is no association between local recurrence and margin distance in lumpectomy resection specimens [25]. However, in our study, a higher rate of recurrence was found in patients who had clear resection margins of lumpectomy specimens (i.e., 75%) (8/24).

This study has few limitations as this was a retrospective, single-institution study. Nevertheless, to the best of our knowledge, no such study has been performed in this part of the world. Therefore, this study does contribute useful data and will have pathway for further prospective studies. Also, the present study is unique in its analysis of the impact of various factors on local recurrence after breast conservation surgery in Asian patients.

## Conclusions

Local disease recurrence after breast conservation surgery can occur in the form of suspicious microcalcifications without an associated mass. The radiologist should be cognizant of the morphological and distribution patterns of malignant calcifications. Moreover, knowledge of various factors that influence the risk of recurrence may also supplement the interpretation of these findings. Stereotactic biopsy should be performed for suspicious calcifications in high-risk patients to ascertain the diagnosis.
